# A non-conventional discontinuous Lagrangian for viscous flow

**DOI:** 10.1098/rsos.160447

**Published:** 2017-02-08

**Authors:** M. Scholle, F. Marner

**Affiliations:** 1 Heilbronn University, Institute for Automotive Technology and Mechatronics, 74081 Heilbronn, Germany; 2 School of Engineering and Computing Sciences, Durham University, Durham DH1 3LE, UK

**Keywords:** potential fields, variational calculus, Navier–Stokes equations, non-equilibrium thermodynamics, analogies, first integrals

## Abstract

Drawing an analogy with quantum mechanics, a new Lagrangian is proposed for a variational formulation of the Navier–Stokes equations which to-date has remained elusive. A key feature is that the resulting Lagrangian is discontinuous in nature, posing additional challenges apropos the mathematical treatment of the related variational problem, all of which are resolvable. In addition to extending Lagrange's formalism to problems involving discontinuous behaviour, it is demonstrated that the associated equations of motion can self-consistently be interpreted within the framework of thermodynamics beyond local equilibrium, with the limiting case recovering the classical Navier–Stokes equations. Perspectives for applying the new formalism to discontinuous physical phenomena such as phase and grain boundaries, shock waves and flame fronts are provided.

## 1. Introduction

For physical systems formulated within the framework of Lagrange's formalism, the dynamics are completely defined by only one function: the Lagrangian. This methodical concept successfully applies to, for example, conservative Newtonian mechanics. Contrary to this, in continuum theories many open problems remain unsolved to this day, especially when considering dissipative processes; the viscous flow of a fluid, given by the Navier–Stokes equations, is a typical example where a formulation by a Lagrangian is missing.

Many attempts at finding a variational formulation of the Navier–Stokes equations have been made: Millikan [1] performed an investigation by assuming a Lagrangian of the form

$$\ell =\ell \left(\text{u},p,\frac{\partial \text{u}}{\partial t},\nabla ⊗\text{u}\right),$$

in terms of the velocity ***u***, the pressure *p* and their first-order derivatives. Despite his rigorous treatment of this inverse problem, his result applies to special flow geometries only. Consequently, in general a different approach is required based on the representation of the observable fields by potentials, i.e. by auxiliary fields representing the observables. For the particular case of inviscid flows, Clebsch [2] was successful in this by means of the potential representation

1.1 u=∇ζ+α∇β,

known as the *Clebsch transformation* [3,4,5]. There are various field theories, for instance Maxwell theory, where a representation of observables in terms of potentials is required in order to establish a proper variational formulation. At first glance, it seems to be a matter of experience that potential fields are required for finding Lagrangians in different field theories. However, in Scholle [6] a convincing explanation is given as to why in continuum theories the use of potentials is absolutely necessary for the construction of a Lagrangian: in order to fulfill the invariance with respect to the full Galilean group, at least one field must be non-measurable and therefore be a potential. In the same paper, a general scheme for Lagrangians is constructed. Using Noether's theorem, canonical formulae give rise to the identification of the relevant observable fields like mass density and flux density, momentum density, stress tensor, energy density and Poynting vector.

It should also be noted that the existence of the Clebsch variables *ζ*,*α*,*β* for an arbitrary velocity field ***u*** can be taken for granted only locally. Their global existence depends on the topological features of the flow; for details we refer, for example, to [7,8]. On the other hand, completeness of the Clebsch representation may be reached by additional pairs of variables. Here we restrict our theory to the classical form (1.1).

Since viscosity leads to dissipation and therefore to the irreversible transfer of mechanical energy to heat, thermal degrees of freedom have to be considered in order to remain consistent to Noether's theorem which implies conservation of energy for systems with time-translation invariance, because otherwise the time-translation invariance would have to be violated by an explicit time-dependence. Seliger & Whitham [9] made a suggestion as to how to embed thermal degrees of freedom in a variational formulation of fluid flow via the Lagrangian

1.2 $$\ell =-\varrho \left[\frac{\partial \zeta }{\partial t}+\alpha \frac{\partial \beta }{\partial t}-s\frac{\partial \vartheta }{\partial t}+\frac{{\text{u}}^{2}}{2}+e(\varrho ,s)\right]$$

and

1.3 u=∇ζ+α∇β−s∇ϑ,

which depends on the specific inner energy *e*(*ϱ*,*s*), given in terms of the mass density *ϱ* and the specific entropy *s*, the three Clebsch potentials *ζ*, *α*, *β* and an additional potential field *ϑ*. The meaning of the latter becomes apparent by calculating the Euler–Lagrange equation with respect to *s*, giving the ‘potential representation’

1.4 $$\left\{\frac{\partial }{\partial t}+\text{u}\cdot \nabla \right\}\vartheta =\frac{\partial e}{\partial s}=T,$$

for the temperature *T*, used three decades previously by Van Dantzig, who termed the field *ϑ* as *thermasy* [10]. Although still restricted to adiabatic and therefore reversible processes, the Lagrangian (1.2) represents a momentous step forward because of the rudimentary embedding of thermodynamics.

By comparing the potential representation (1.3) with the one proposed by Clebsch (1.1) for the isothermal case, it becomes apparent that any kind of extension of the system, by additional degrees of freedom as well as by additional physical effects, requires an adjustment of the potential representation; see, for example, Wagner [11]. Scholle [6] provides a general explanation for the necessary use of different potential representations of the observables for different physical systems along the lines of a rigorous analysis of the fundamental symmetries the Lagrangian has to fulfil, with particular regard to Galilean invariance. In the same paper, an easily manageable symmetry criterion for verifying Galilean invariance is derived. Based on these preliminary findings, Scholle [12,13] suggested a Lagrangian for viscous flow by supplementing the Lagrangian (1.2) with additional terms leading to partial success: on the one hand, the phenomenon ‘viscosity’ occurs in a qualitatively correct manner, as demonstrated by three simple flow examples. On the other hand, the equations of motion resulting from the variation of Hamilton's principle differ from the Navier–Stokes equations and, therefore, their solutions reveal notable quantitative differences to those of the latter. Similar experiences are reported by Zuckerwar & Ash [14], who made an analogous suggestion for a Lagrangian considering volume viscosity in particular.

Despite this partial success, the need to improve the existing approach is obvious in order to obtain solutions from Hamilton's principle suitable for relevant flow problems. In this article, an innovative idea by Anthony [15] is applied, based on the reformulation of the Lagrangian in terms of complex fields. This can also be understood as the inversion of Madelung's idea [16] of reformulating the complex Schrödinger's equation into a hydrodynamic form.

## 2. Construction of the Lagrangian

First, as demonstrated in prior work [17,18,12,13], Seliger and Whitham's Lagrangian (1.2) can be re-written alternatively as

2.1 $$\ell =-\varrho \left[D_{t}\zeta +\alpha D_{t}\beta -sD_{t}\vartheta -\frac{{\text{u}}^{2}}{2}+e(\varrho ,s)\right],$$

in terms of the extended set of independent fields *ψ*=(***u***,*ζ*,*α*,*β*,*ϱ*,*s*,*ϑ*) and their material time derivatives

2.2 $$D_{t}=\frac{\partial }{\partial t}+\text{u}\cdot \nabla .$$

The above form of the Lagrangian yields two benefits: first, the potential representation (1.3) of the velocity field results from a variation with respect to ***u*** and hence does not need to be prescribed; second, by adding terms to the Lagrangian depending on first-order derivatives of ***u*** in order to consider friction, the extended Lagrangian remains of first order. The latter is a useful feature because otherwise, i.e. in the case of a Lagrangian containing second-order derivatives of the fields, the computation of (i) the corresponding Euler–Lagrange equations and (ii) the canonical densities and flux densities resulting from Noether's theorem become more complicated. It is also very useful to avoid derivatives of order higher than one when applying Ritz's direct method to problems formulated in curvilinear coordinates.

### 2.1 Conventional approach and examples

In this section, the basic ideas and relevant findings in Scholle [19,12,13] are revisited in a concise form in order to capture the present state of the theory and the associated open problems.

For the extension of the Lagrangian (2.1) to incorporate viscosity, it seems reasonable to simply add terms modifying the entropy balance resulting from variation with respect to the thermasy *ϑ* as

2.3 $$-\frac{\partial }{\partial t}(\varrho s)-\nabla \cdot (\varrho s\text{u})=0,$$

the homogeneity of this expression indicating that only adiabatic processes are considered in (2.1). Note that the above balance alternatively results from Noether's theorem with respect to the transformation *ϑ*→*ϑ*+*ε* with *ε*=const., which is a symmetry transformation of the Lagrangian (2.1). In order to take into account the production of entropy, the symmetry with respect to the transformation *ϑ*→*ϑ*+*ε* has to be broken, which is achieved in the easiest way by adding a term linearly dependent on *ϑ*, i.e. *ϑϕ*_*d*_/*T*, to the Lagrangian, where the dissipation heat *ϕ*_*d*_ should be positive and depend on the spatial derivatives of the velocity as the primary cause for the physical phenomenon ‘viscosity’. Both are satisfied by assuming for *ϕ*_*d*_ a quadratic dependence on ∂_*j*_*u*_*i*_, according to the classic literature on viscous flow [20,21]. Finally, via the factor 1/*T* the character of the entropy as ‘weighted heat’, according to *δQ*=*T*d*S*, is accounted for. The above considerations provide the motivation behind the following extended Lagrangian [12,13]:

2.4 $$\ell =-\varrho \left[D_{t}\zeta +\alpha D_{t}\beta -sD_{t}\vartheta -\frac{{\text{u}}^{2}}{2}+e(\varrho ,s)\right]+\frac{\vartheta }{T}\left[\eta \;\mathrm{tr}{\underline{D}}^{2}+\frac{\eta^{\prime }}{2}{\left(\nabla \cdot \text{u}\right)}^{2}\right],$$

where *η* is the shear viscosity, *η*′ the volume viscosity of the fluid and

2.5 $$\underline{D}:=\frac{1}{2}[\nabla ⊗\text{u}+{\left(\nabla ⊗\text{u}\right)}^{t}]$$

is the tensor of the shear rate; *tr* denotes the trace of a tensor. The temperature *T*, according to classical thermodynamics, is given by

2.6 $$T=\frac{\partial e}{\partial s}.$$

Note that neither an external force nor heat conduction is considered here. Now, by variation with respect to the thermasy *ϑ*, the equation

2.7 $$\frac{\partial }{\partial t}(\varrho s)+\nabla \cdot (\varrho s\text{u})=\frac{\eta }{T}\;\mathrm{tr}{\underline{D}}^{2}+\frac{\eta^{\prime }}{2T}{\left(\nabla \cdot \text{u}\right)}^{2}$$

results as an entropy balance with an entropy production rate on the right-hand side due to dissipation, as expected. Furthermore, the above Lagrangian fulfils the necessary symmetry requirements for Galilean invariance, as analysed in detail in §A.1 of appendix A; however, an unexpected feature transpires. The momentum density ***p***, resulting as a canonical Noether observable, does not equal the mass flux density *ϱ****u***. The difference between both

2.8 $${\text{p}}^{*}:=\text{p}-\varrho \text{u}=-2\eta \nabla \cdot \left(\frac{\vartheta }{T}\underline{D}\right)-\eta^{\prime }\nabla \left(\frac{\vartheta }{T}\nabla \cdot \text{u}\right),$$

termed *quasi-momentum density*, needs to be explained physically. According to Scholle [6], p* could be due to contributions to the system's momentum balance beyond the scope of the continuum hypothesis on a molecular scale, e.g. Brownian motion. This question is discussed in more detail in §5. Regardless, the dynamics induced by the Lagrangian (2.4) go beyond the scope of classical theory, the resulting equations of motion differing significantly from the Navier–Stokes equations. In the case of incompressible flow and negligible buoyancy they read [12,13,19]

2.9 $$\begin{array}{rl} D_{t}\text{u} & =-\frac{\nabla p}{\varrho_{0}}+\nu \{D_{t}+\nabla ⊗\text{u}\}\left[2\underline{D}\nabla \left(\frac{\vartheta }{T}\right)+\frac{\vartheta }{T}\Delta \text{u}\right]-\nu \;\mathrm{tr}{\underline{D}}^{2}\nabla \left(\frac{\vartheta }{T}\right), \end{array}$$

2.10 $$\begin{array}{rl} \nabla \cdot \text{u} & =0 \end{array}$$

2.11 $$\begin{array}{rl} \text{and}\;D_{t}\left(\frac{\vartheta }{T}\right) & =1, \end{array}$$

with constant mass density *ϱ*=*ϱ*_0_ and kinematic viscosity *ν*:=*η*/*ϱ*_0_. Looking at the above set of PDEs, two striking features immediately become apparent, namely:

(i) the resulting field equations are third-order PDEs, not second-order ones like the Navier–Stokes equations;
(ii) the thermasy *ϑ* appears explicitly in the field equations as an additional degree of freedom.

**Figure 1.**
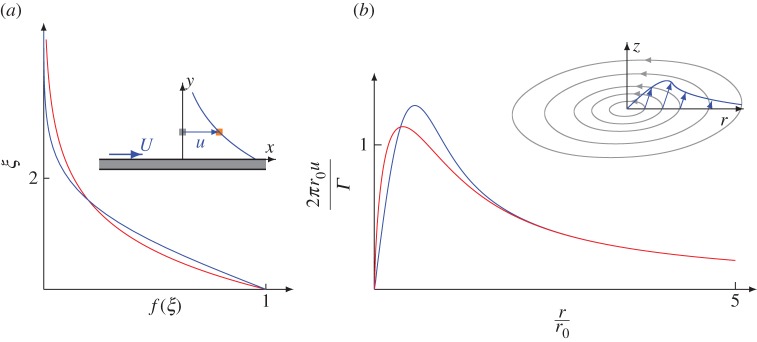
Resulting velocity profiles for (*a*) the flow over a suddenly moving plate and (*b*) the Lamb–Oseen vortex (at time $t=\pi \varrho r_{0}^{2}/25\eta$). For both examples, the solution resulting from the equations of motion (2.9)–(2.11) (red) are compared to the respective profile resulting from the original Navier–Stokes equations (blue).

The above qualitative features have also been found in the case of compressible flow with pure volume viscosity by Zuckerwar & Ash [14], and the appearance of an additional physically relevant degree of freedom, in particular, appears also in the variational formulation of heat conduction proposed by Anthony [15] and is interpreted by him as a measure for the *deviation from the thermodynamical local equilibrium*. Similar assumptions are made in Zuckerwar & Ash [14]. At first glance, there seems to be a realistic chance to interpret the additional terms and degrees of freedom in the above evolution equations (2.7), (2.9)–(2.11) as an extension of the classical theory towards non-equilibrium thermodynamics. In order to test this hypothesis, three ‘benchmark tests’ have been performed [12,13]:

(i) Plane Couette flow, i.e. shear flow between two parallel plates, the upper one of which is moving with a constant speed *U*, representing one of the most prominent examples in fluid dynamics. Considering the unidirectional flow ***u***=*u*(*y*)***e***_*x*_ with boundary conditions *u*(0)=0 and *u*(*h*)=*U*, a solution of equations (2.9)–(2.11) is given by the linear velocity profile *u*(*y*)=*Uy*/*h* and a constant pressure *p* in full accordance with the Navier–Stokes equations.
(ii) As an example of a transient flow, a suddenly moving plate is considered: a horizontal plate of infinite extension is covered by a fluid at rest at *t*<0, see figure 1*a*. At *t*=0 the plate suddenly starts moving with constant velocity *U* in the horizontal direction, invoking a flow inside the fluid. The initial conditions, *t*_0_=0 and *u*_0_=0, have to be considered. Since no characteristic length is contained in this problem, a representation of the velocity profile in terms of a similarity variable ***u***=*Uf*(*ξ*) with $\xi =y/\sqrt{\nu t}$ [20] is used, leading to the solution f(ξ)=exp⁡(−ξ) of the equations of motion (2.9)–(2.11), whereas the classical solution of the Navier–Stokes equations reads *f*(*ξ*)=1−*erf*(*ξ*/2) [20] involving the error function *erf*. In figure 1*a*, both resulting profiles are compared to each other, revealing qualitatively concordant profiles with quantitative differences.
(iii) The Lamb–Oseen vortex [3], the flow geometry of which is shown in figure 1*b*, is another example of a transient flow: using cylindrical coordinates *r*,*φ*,*z*, a similarity variable $\xi :=r/\sqrt{\nu t}$ is available allowing for a representation of the velocity as ***u***=*u*(*r*,*t*)***e***_*φ*_ with the time-depending profile *u*(*r*,*t*)=*Γf*(*ξ*)/(2*πr*) for a given circulation *Γ*. According to Scholle [12,13,19], from (2.9)–(2.11) the analytical form *f*(*ξ*)=1−*ξK*_1_(*ξ*) is obtained with the modified Bessel function of first order, *K*_1_. In contrast, $f(\xi )=1-\exp(-\xi^{2}/4)$ occurs in the classical solution [3]. Both solutions are compared in figure 1*b*, revealing again qualitative accordance with quantitative differences.

Note that for the above three examples (i)–(iii), *ϑ*/*T*=*t* has been used as a particular solution of the evolution equation (2.11), leading to the simplified form

**Figure 2.**
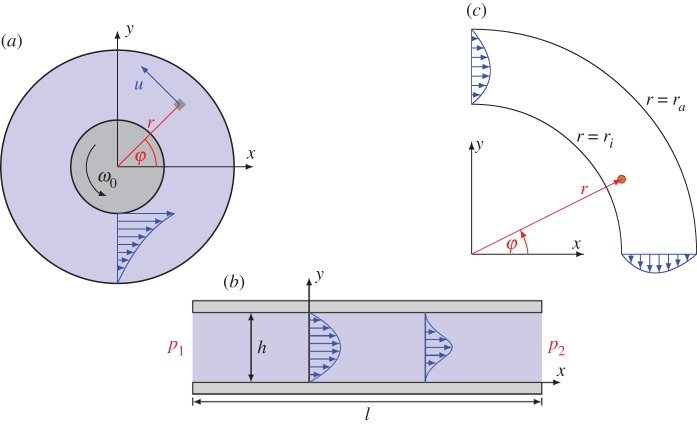
Flow geometry of the three flows: (*a*) Taylor–Couette flow, (*b*) plane Poiseuille flow and (*c*) Poiseuille flow in a curved channel. The velocity profiles resulting from the equations of motion are included.

2.12 $$D_{t}\text{u}=-\frac{1}{\varrho_{0}}\nabla p+\nu \{D_{t}+\nabla ⊗\text{u}\}\left[\frac{\vartheta }{T}\Delta \text{u}\right]$$

of the equations of motion (2.9). Summarizing the above examples, it can be ascertained that the phenomenon of viscosity is at least qualitatively captured by Hamilton's principle. Hence, the Lagrangian (2.4) reflects a relevant step forward to a satisfying description of viscous flow within the framework of Lagrange formalism. On the one hand, the differences between the classical theory and the results obtained by variation are quite pronounced for the transient flow examples (ii) and (iii). It is open to dispute if they can be explained as non-equilibrium effects. On the other hand, alternative equations of motion containing additional terms related to thermal relaxation are discussed in the literature; for instance, we refer to Ash & Zardadkhan [22,23] who made use of the Navier–Stokes equations supplemented with a ‘pressure relaxation term’, implying a vortex solution considerably different from the classical Lamb–Oseen vortex in order to resolve discrepancies between existing models and observations on dust devils and tornados. Apart from the examples reported in Scholle [12,13,19], we consider three further examples in order to get things straight concerning the question of whether the non-classical form of the equations of motion (2.9)–(2.11) can consistently be explained as being due to non-equilibrium thermodynamics. These are:

(iv) The Taylor–Couette flow between two cylinders of radius *r*_*i*_ and *r*_*a*_, see figure 2*a*, invoked by a rotation of the inner cylinder with angular velocity *ω*_0_, is characterized by a flow geometry ***u***=*u*(*r*)***e***_*φ*_. By applying this to the equations of motion (2.10) and (2.12) and considering the boundary conditions *u*(*r*_*i*_)=*ω*_0_*r*_*i*_ and *u*(*r*_*a*_)=0, we obtain as a solution
  $$u(r)=\frac{\omega_{0}r_{i}^{2}r_{a}}{r_{a}^{2}-r_{i}^{2}}\left[\frac{r_{a}}{r}-\frac{r}{r_{a}}\right]$$
  and
  $$p(r)=\varrho \int \frac{u{\left(r\right)}^{2}}{r}\;dr,$$
  in perfect agreement with classical theory.
(v) Plane Poiseuille flow between two parallel plates, a distance *h* apart, driven according to figure 2*b* by a pressure gradient (*p*_1_−*p*_2_)/*l*, with a unidirectional flow geometry ***u***=*u*(*y*)***e***_*x*_ assumed. Considering no-slip conditions *u*(0)=*u*(*h*)=0 at the lower and upper plate, the solution of the equations of motion (2.10), (2.12) reads
  2.13 $$u(y)=\frac{Ky(h-y)}{2}$$
  and
  2.14 $$p(x,y,t)=p_{1}+\eta K[x+u(y)t].$$
  By identifying *K*=(*p*_1_−*p*_2_)/(*ηl*), the above velocity profile (2.13) is in perfect agreement with the classical solution [20]. However, the associated pressure (2.14) contains an additional term *ηKu*(*y*)*t*, by which the adherence of the boundary conditions for the pressure *p* at the inflow and the outflow is inhibited. Moreover, the pressure is unsteady and, as a non-physical feature, it tends to infinity with increasing time. The reason for the latter problem stems from the choice of the particular thermasy solution *ϑ*/*T*=*t* of the evolution equation (2.11) which is increasing with time as well, a fact that seems to be problematic not only for this specific flow problem but for problems in fluid mechanics in general, as stated in Scholle [24]. In their response to the comment [24], Zuckerwar and Ash [25] suggested to construct a time-independent solution of the evolution equation (2.11), fulfilling ***u***⋅∇(*ϑ*/*T*)=1. Following their suggestion, as a steady solution for the weighted thermasy the expression
  2.15 $$\frac{\vartheta }{T}=\frac{x}{u(y)}+f_{1}(y)$$
  is obtained with arbitrary integration function *f*_1_(*y*), see appendix A.2. The associated solution of the equations of motion (2.9) and (2.10) reads, according to appendix A.2, implicitly as
  2.16 $$\begin{array}{rl} y & ={\sqrt{\frac{2u}{K}}}_{2}F_{1}\left(\frac{1}{6},\frac{1}{2};\frac{7}{6};\frac{u^{3}}{u_{\text{max}}^{3}}\right), \end{array}$$
  2.17 $$\begin{array}{rl} u_{\text{max}} & =\frac{9\Gamma {\left(\frac{2}{3}\right)}^{2}\Gamma {\left(\frac{5}{6}\right)}^{2}}{8\pi^{3}}Kh^{2} \end{array}$$
  2.18 $$\begin{array}{rl} \text{and}\;p & =p_{0}-\eta Kx+\eta \frac{\vartheta }{T}\frac{u^{\prime }{\left(y\right)}^{2}}{2}, \end{array}$$
  with the Gaussian hypergeometric function _2_*F*_1_. The velocity profile (2.17) is visualized in figure 2*b* (right) and differs markedly from the classical parabolic profile (2.13) (left), especially due to the fact that its first-order derivative vanishes at the walls, *u*′(0)=*u*′(*h*)=0, thus indicating a zero wall shear stress. As another conspicuous feature, the *y*-dependence of the pressure inhibits again fulfilment of the boundary conditions for the pressure *p* at the inflow and the outflow. In summary, no physically meaningful steady solution of (2.9)–(2.11) can be constructed for a plane Poiseuille flow.
(vi) Poiseuille flow in a curved channel, see figure 2*c*, as investigated by Richter [26], who discovered qualitatively the same problems known from the plane Poiseuille flow. In particular, the resulting pressure solution,
  $$p=2\frac{p_{2}-p_{1}}{\pi }\left[\varphi +\frac{u(r)t}{r}\right]+\varrho \int \frac{u{\left(r\right)}^{2}}{r}\;dr,$$
  is found to similarly contain a non-physical term increasing with time. Other attempts to find a time-independent solution matching the boundary conditions have remained unsuccessful, as in the case of the previous example.

Summarizing the above benchmark tests, we conclude that only for two of the six examples, namely the shear-driven flows (i) and (iv), are the classical solutions recovered. For the two transient flows, (ii) and (iii), reasonable solutions of the field equations and boundary conditions have been found, with quantitatively different velocity profiles compared to the classical solutions. For the two pressure-driven flows, (v) and (vi), no adequate solutions of the field equations could be constructed which simultaneously fulfil the pressure boundary conditions. Hence, the variational principle based on the Lagrangian (2.4) does not recover the dynamics of viscous flow in a proper way, since its applicability seems to be restricted to special flow problems only.

Moreover, four of the six benchmark solutions contradict the hypothesis that the differences between them and classical theory can be explained by effects beyond the scope of thermodynamic equilibrium: apart from the non-physical features discovered above, one would expect that the non-equilibrium solution tends towards the classical equilibrium solution if a special relaxation parameter in the problem, physically related to the deviation from equilibrium, tends to infinity. This is not the case here since no additional parameters exist but mass density, viscosity and specific heat.

Analysing the above examples in more detail, the explicit appearance of the weighted thermasy *ϑ*/*T* in the equations of motion (2.12) seems to be the crucial issue leading to non-physical solutions, since *ϑ*/*T* turns out to be of unlimited growth, either spatially or temporally, which also prohibits its interpretation in connection with non-equilibrium thermodynamics. Moreover, the anomalous relation (2.8) between mass flux density and momentum density implies that the discrepancy between mass flux and momentum also tends to increase spatially or temporally due to the *ϑ*/*T*-dependence.

A modification of the Lagrangian (2.4) is provided below which overcomes the above-highlighted anomalies.

### 2.2 Alternative approach based on complex fields

In 1927, Madelung [16] discovered a remarkable analogy between quantum mechanics and fluid mechanics by reformulating the complex Schrödinger's equation into a hydrodynamic form: by decomposing the quantum mechanical state function *ψ* into modulus and phase according to

2.19 $$\psi =\sqrt{\frac{\varrho }{m}}\exp\left(-i\frac{m}{\hbar }\Phi \right),$$

Schrödinger's equation is transformed to the set of PDEs

2.20 $$\frac{\partial \varrho }{\partial t}+\nabla \cdot (\varrho \nabla \Phi )=0$$

and

2.21 $$\frac{\partial \Phi }{\partial t}+\frac{1}{2}{\left(\nabla \Phi \right)}^{2}-\frac{\hbar }{4\pi m^{2}}\frac{\Delta \varrho }{\varrho }+\frac{U}{m}=0.$$

These are obviously the equations of motion of a kind of fluid, the so-called *Madelung fluid*: equation (2.20) is the continuity equation and (2.21) is Bernoulli's equation for a fluid with the ‘unusual’ pressure function $P=-\hbar \Delta \varrho /(4\pi m^{2}\varrho )$ and with vorticity-free velocity field ***u***=∇*Φ*. Based on these substitutions, Madelung established a fluid mechanics picture of Schrödinger's theory.

Many years later, Anthony [15] suggested the reverse of this idea, i.e. form a ‘Schrödinger-picture’ of fluid mechanics and thermodynamics, by combining the density *ϱ* and the Clebsch variable *ζ* in (1.2) to form a complex *matter field* *ψ* according to (2.19). Moreover, he introduced two more complex fields, namely a complex vortex potential *Ω* by combining the two remaining Clebsch variables *α*,*β* and the complex field of thermal excitation *χ*, giving the temperature by its absolute square: $T=\bar{\chi }\chi$. The motivation for this transformation is originally given by Anthony's entropy concept: the entropy balance results from a canonical procedure related to the phase translation invariance of the complex fields as a balance of second kind within the framework of second variation and related stability criteria. Furthermore, Anthony states that by the complex representation a basic concept is given for an accurate formulation of thermodynamics of irreversible processes within the framework of Lagrange formalism.

For convenience, we only apply a partial transformation to complex fields in a slightly modified form to the Lagrangian (2.4): by introducing *T*_0_ as a constant reference temperature and *c*_0_ as a reference constant for the specific heat and considering the identity

$$-sD_{t}\vartheta =D_{t}[(c_{0}-s)\vartheta ]-c_{0}T_{0}\exp\left(\frac{s}{c_{0}}\right)D_{t}\left[\exp\left(-\frac{s}{c_{0}}\right)\frac{\vartheta }{T_{0}}\right],$$

motivation is given for the generalized definition of the field of thermal excitation as

2.22 $$\chi :=\sqrt{c_{0}T_{0}}\exp\left(\frac{s}{2c_{0}}-i\omega_{0}\exp\left(-\frac{s}{c_{0}}\right)\frac{\vartheta }{T_{0}}\right),$$

supplemented by the substitution

2.23 $$\Phi :=\zeta +(c_{0}-s)\vartheta ,$$

for the Clebsch variable *ζ*. Note that in (2.22) another constant, *ω*_0_, has been introduced due to dimensional reasons, like *T*_0_ and *c*_0_ before. Although there is no general rule how the three constants *T*_0_,*c*_0_ and *ω*_0_ have to be chosen, it is reasonable to choose the reference temperature *T*_0_ as a ‘typical’ temperature and *c*_0_ as a ‘typical’ specific heat. In the case of an incompressible flow with constant specific heat, discussed subsequently in §(c), the choice of *c*_0_ is obvious and the choice of *T*_0_ is arbitrary since the resulting Lagrangian (2.31) does not depend on it any more. By contrast, the choice of *ω*_0_ is not obvious, nor how the physics is affected by it. This will be analysed and discussed carefully in the following.

On substituting for (2.22) and (2.23), it follows that

2.24 Dtζ−sDtϑ=DtΦ+1ω0Im(χ¯Dtχ)

and

**Figure 3.**
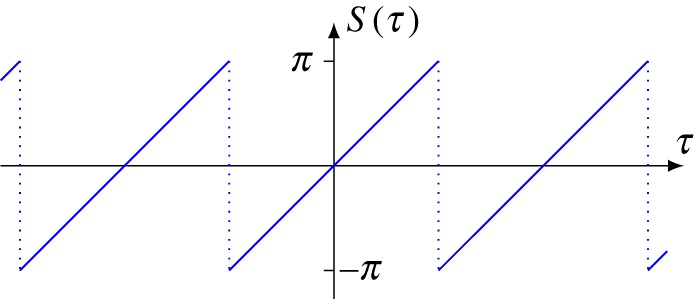
The sawtooth function.

2.25 $$S\left(\omega_{0}\exp\left(-\frac{s}{c_{0}}\right)\frac{\vartheta }{T_{0}}\right)=-\arg\chi =-i\;\ln\;\sqrt{\frac{\bar{\chi }}{\chi }},$$

with the sawtooth function (see also figure 3)

2.26 $$S(x):=x-2\pi \left\lfloor \frac{x+\pi }{2\pi }\right\rfloor .$$

While the first equation (2.24) allows for a unique transformation of the respective real-valued terms in the Lagrangian (2.4) into terms of the complex thermal excitation field, the second equation (2.25) reveals that no equivalent for the thermasy *ϑ* explicitly appearing in the friction term of (2.4) can be constructed in terms of the complex field *χ*. The reason for this is the non-uniqueness of the argument of a complex number. The obviously most feasible way to resolve this issue is the use of

$$\frac{T_{0}}{i\omega_{0}}\exp\left(\frac{s}{c_{0}}\right)\;\ln\;\sqrt{\frac{\bar{\chi }}{\chi }}=\frac{\bar{\chi }\chi }{i\omega_{0}c_{0}}\;\ln\;\sqrt{\frac{\bar{\chi }}{\chi }},$$

as a substitute for *ϑ*, leading to the modified Lagrangian

2.27 ℓ=−ϱ[DtΦ+αDtβ+1ω0Im(χ¯Dtχ)−u22+e]+χ¯χ ln ⁡χ¯χiω0c0T[ηtrD_2+η′2(∇⋅u)2].

Comparing this Lagrangian with (2.4), two remarkable differences are discernible: first, the modified Lagrangian is discontinuous due to the logarithmic term; second, the angular frequency *ω*_0_, which has primarily been introduced for dimensional reasons, becomes a relevant parameter, the physical meaning of which will be clarified subsequently. The most striking feature, however, is that the unlimited weighted thermasy *ϑ*/*T* appearing in (2.4) has been replaced by an expression with limited values between −*π* and *π*.

### 2.3 Incompressible flow with constant specific heat and external force

We consider a fluid flow with constant mass density, constant specific heat and without thermal expansion:

2.28 $$\begin{array}{rl} \varrho & =\varrho_{0}, \end{array}$$

2.29 $$\begin{array}{rl} s & =c_{0}\;\ln\;\left(\frac{T}{T_{0}}\right) \end{array}$$

2.30 $$\begin{array}{rl} \text{and}\;e & =c_{0}T. \end{array}$$

Note that (2.29) implies $\bar{\chi }\chi =c_{0}T$ for the field of thermal excitation (2.22) in accordance with Anthony [15]. Furthermore, since for the incompressible case volume viscosity is excluded from the very beginning, the term with *η*′ in (2.27) is absent. Finally, we add the specific potential energy *V* =*V* (***x***,*t*) of an external force, leading to the simplified Lagrangian:

2.31 ℓ=−ϱ0[DtΦ+αDtβ+1ω0 Im(χ¯Dtχ)−u22+χ¯χ+V−νiω0 ln ⁡χ¯χ trD_2].

## 3. Variation with discontinuous Lagrangian: general formalism

In conventional variational calculus, Euler–Lagrange equations can be computed if the Lagrangian is two times continuously differentiable [27]. If this basic requirement is not fulfilled, a non-standard approach is required for variation, which is developed in the following.

We consider a variational principle *δI*=0 where *I* is given by

3.1 $$I=\int_{t_{1}}^{t_{2}}{∭}_{V}\ell (\psi_{i},{\dot{\psi }}_{i},\nabla \psi_{i})\;dV\;dt,$$

depending on independent fields *ψ*_*i*_ with *i*=1,…,*N* and *ψ*_*N*_=*φ*. The Lagrangian ℓ is assumed to be discontinuous with respect to *φ* at fixed values *φ*_*n*_, *n*=1,…,*N*_*S*_, but continuously differentiable with respect to all other fields, *ψ*_*i*_ with *i*<*N*, and also continuously differentiable with respect to all derivatives of any field, including $\dot{\varphi }$ and ∇*φ*. In three-dimensional space, the discontinuities with respect to *φ* become manifest along surfaces *S*_*n*_(*t*) defined by

3.2 $$S_{n}:=\{\text{x}\;|\;\varphi (\text{x},t)=\varphi_{n}\},\;n=1,\ldots ,N_{S},$$

intersecting the system's volume *V* into a finite number *N*_*S*_+1 of sub-volumes according to:

$$V=\sum_{n=0}^{N_{S}}V_{n},$$

where the sub-volume *V*_*n*_ denotes the region between *S*_*n*_ and *S*_*n*+1_ apart from *V*_0_ and *V*_*N*_*S*__ denoting the region between the system's boundary ∂*V* and *S*_1_ or *S*_*N*_*S*__, respectively.

**Figure 4.**
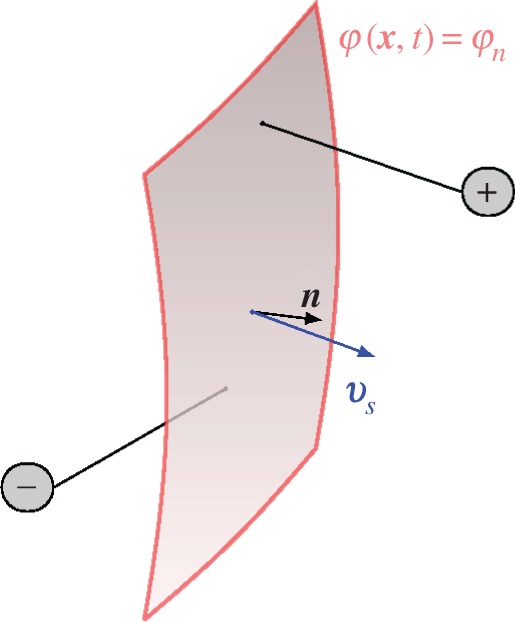
Surface *S*_*n*_, along which a discontinuity becomes manifest.

From the physical viewpoint, these time-dependent interfaces, *S*_*n*_, may be related to any kind of discontinuous phenomena like phase boundaries between non-mixable fluids, propagating shock fronts in gaseous media or flame fronts. Their local propagation velocity is denoted by ***v***_*s*_ and the vector normal to the surface by ***n*** (see figure 4); the orientation of ***n*** is defined by the convention ***n***⋅***v***_*s*_>0.

### 3.1 Euler–Lagrange equations

First, only the subset of variations *δψ*_*i*_=0 is considered with *δψ*_*i*_=0 at the interfaces *S*_*n*_ and at the system's boundary ∂*V* . Free variation is assumed inside the sub-volumes *V*_*n*_. Note that this kind of variation does not cause any shift of the interfaces *S*_*n*_. Under these assumptions, the usual derivation procedure leading to the Euler–Lagrange equations can be performed separately inside each sub-volume, and hence the well-known Euler–Lagrange equations [27],

3.3 $${\mathrm{EL}}_{i}:=\frac{\partial \ell }{\partial \psi_{i}}-\frac{\partial }{\partial t}\left(\frac{\partial \ell }{\partial {\dot{\psi }}_{i}}\right)-\nabla \cdot \left(\frac{\partial \ell }{\partial \nabla \psi_{i}}\right)=0,$$

remain valid piecewise at each sub-volume *V*_*n*_.

### 3.2 Matching conditions

Next, a larger set of variations with *δψ*_*i*_≠0 at the interfaces *S*_*n*_ is considered for *i*=1,…,*N*−1, but the variation of *ψ*_*N*_=*φ* is again restricted to *δφ*=0 in order to exclude any shift of the interfaces themselves. Like before, *δψ*_*i*_=0 is again prescribed at the system's boundary. Now, variation with respect to *ψ*_1_,…,*ψ*_*N*−1_ leads to

3.4 $$\begin{array}{rl} \delta I & =\int_{t_{1}}^{t_{2}}\sum_{n=0}^{N_{S}}{∭}_{V_{n}}\left[\frac{\partial \ell }{\partial \psi_{i}}\delta \psi_{i}+\frac{\partial \ell }{\partial {\dot{\psi }}_{i}}\delta {\dot{\psi }}_{i}+\frac{\partial \ell }{\partial \nabla \psi_{i}}\nabla \delta \psi_{i}\right]dV\;dt,\\ & =\int_{t_{1}}^{t_{2}}\sum_{n=0}^{N_{S}}{∭}_{V_{n}}\left[{\mathrm{EL}}_{i}\delta \psi_{i}+\frac{\partial }{\partial t}\left(\frac{\partial \ell }{\partial {\dot{\psi }}_{i}}\delta \psi_{i}\right)+\nabla \cdot \left(\frac{\partial \ell }{\partial \nabla \psi_{i}}\delta \psi_{i}\right)\right]dV\;dt, \end{array}$$

using the abbreviation in (3.3) for the Euler–Lagrange expressions. By means of the Gaussian theorem

$${∭}_{V_{n}}\nabla \cdot \left(\frac{\partial \ell }{\partial \nabla \psi_{i}}\delta \psi_{i}\right)dV=\underset{\partial V_{n}}{\text{∯}}\text{n}\cdot \frac{\partial \ell }{\partial \nabla \psi_{i}}\delta \psi_{i}\;dS,$$

and Reynold's transport theorem well-known from fluid dynamics [20]

$${∭}_{V_{n}}\frac{\partial }{\partial t}\left(\frac{\partial \ell }{\partial {\dot{\psi }}_{i}}\delta \psi_{i}\right)dV=\frac{d}{dt}{∭}_{V_{n}}\frac{\partial \ell }{\partial {\dot{\psi }}_{i}}\delta \psi_{i}\;dV-\underset{\partial V_{n}}{\text{∯}}\text{n}\cdot {\text{v}}_{s}\frac{\partial \ell }{\partial {\dot{\psi }}_{i}}\delta \psi_{i}\;dS,$$

with ***v***_*s*_ being the velocity of the propagating interface *S*_*n*_ (figure 4), the variation takes the form

3.5 $$\begin{array}{rl} \delta I & =\int_{t_{1}}^{t_{2}}\sum_{n=0}^{N_{S}}{∭}_{V_{n}}{\mathrm{EL}}_{i}\delta \psi_{i}\;dV\;dt+\sum_{n=0}^{N_{S}}{\frac{d}{dt}{∭}_{V_{n}}\frac{\partial \ell }{\partial {\dot{\psi }}_{i}}\delta \psi_{i}\;dV|}_{t_{1}}^{t_{2}}\\ & \;+\int_{t_{1}}^{t_{2}}\sum_{n=0}^{N_{S}}\underset{\partial V_{n}}{\text{∯}}\text{n}\cdot \left[\frac{\partial \ell }{\partial \nabla \psi_{i}}-{\text{v}}_{s}\frac{\partial \ell }{\partial {\dot{\psi }}_{i}}\right]\delta \psi_{i}\;dS\;dt. \end{array}$$

Since *δψ*_*i*_=0 at ∂*V*_*n*_ and at the initial and final time *t*_1,2_, the above identity simplifies, making use of the Euler–Lagrange equations (3.3), to

$$\delta I=\int_{t_{1}}^{t_{2}}\sum_{n=0}^{N_{S}}\underset{\partial V_{n}}{\text{∯}}\text{n}\cdot \left[\frac{\partial \ell }{\partial \nabla \psi_{i}}-{\text{v}}_{s}\frac{\partial \ell }{\partial {\dot{\psi }}_{i}}\right]\delta \psi_{i}\;dS\;dt.$$

In the following, the limit of the respective discontinuous expression by approaching it from the front side (subscript +) or the back side (subscript −) of the interface *S*_*n*_ is indicated by [⋯ ]_±_. Front and back side are defined by the orientation of the normal vector ***n*** according to figure 4. Then, by decomposing each surface integral over ∂*V*_*n*_ in one integral along the front side of *S*_*n*_ and another one along the back side of *S*_*n*+1_, we find in general

3.6 $$\begin{array}{rl} & \sum_{n=0}^{N_{S}}\underset{\partial V_{n}}{\text{∯}}\text{n}\cdot [\cdots ]\delta \psi_{i}\;dS=\sum_{n=1}^{N_{S}-1}\left[\iint_{S_{n+1}}\text{n}\cdot {\left[\cdots \right]}_{-}\delta \psi_{i}\;dS-\iint_{S_{n}}\text{n}\cdot {\left[\cdots \right]}_{+}\delta \psi_{i}\;dS\right]\\ & \;+\iint_{S_{1}}\text{n}\cdot {\left[\cdots \right]}_{-}\delta \psi_{i}\;dS-\iint_{S_{N_{S}}}\text{n}\cdot {\left[\cdots \right]}_{+}\delta \psi_{i}\;dS=\sum_{n=1}^{N_{S}}\iint_{S_{n}}\text{n}\cdot ({\left[\cdots \right]}_{-}-{\left[\cdots \right]}_{+})\delta \psi_{i}\;dS, \end{array}$$

and, in particular,

$$\delta I=\int_{t_{1}}^{t_{2}}\sum_{n=1}^{N_{S}}\iint_{S_{n}}\text{n}\cdot \left[[\frac{\partial \ell }{\partial \nabla \psi_{i}}-{\text{v}}_{s}\frac{\partial \ell }{\partial {\dot{\psi }}_{i}}]\right]\delta \psi_{i}\;dS\;dt,$$

where the double square bracket indicates the jump at the interface: $[[\ldots ]]:={\left[\cdots \right]}_{-}-{\left[\cdots \right]}_{+}$. Thus, variation *δI*=0 delivers

3.7 $$\text{n}\cdot \left[[\frac{\partial \ell }{\partial \nabla \psi_{i}}-{\text{v}}_{s}\frac{\partial \ell }{\partial {\dot{\psi }}_{i}}]\right]=0,$$

as *matching conditions* for the generalized fluxes, *i*=1,…,*N*−1 at each interface.

Independent of the formal proof given above the matching conditions can also be understood as *natural boundary conditions* at the phase boundaries in a multiphase flow when assuming that all phases of the flow consist of the same liquid, leading to the same equation (3.7).

### 3.3 Production condition

**Figure 5.**
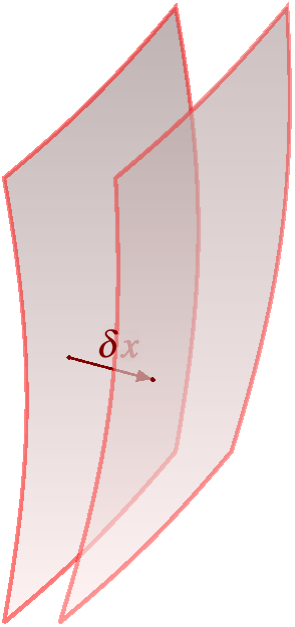
Variation of an interface caused by variation of *φ*.

Finally, *δψ*_*i*_=0 is again prescribed at the system's boundary, but apart from this free variation of all fields is allowed overall inside *V* , including free variation of *ψ*_*N*_=*φ*. As a consequence of the latter, the position of the interfaces *S*_*n*_ is varied too, see figure 5: an arbitrary point ***x*** of *S*_*n*_ defined by (3.2) is shifted to a different position ***x***+*δ****x*** according to

$$\begin{array}{rl} \varphi_{n} & =\varphi (\text{x}+\delta \text{x},t)+\delta \varphi (\text{x}+\delta \text{x},t)=\varphi (\text{x},t)+\delta \text{x}\cdot \nabla \varphi (\text{x},t)+\delta \varphi (\text{x},t)+O(\delta {\text{x}}^{2})\\ & =\varphi_{n}+\delta \text{x}\cdot \nabla \varphi (\text{x},t)+O(\delta {\text{x}}^{2}), \end{array}$$

leading to the identity:

3.8 δx⋅∇φ=−δφ,

for the local shift *δ****x*** of the discontinuity interface *S*_*n*_. In the case that *S*_*n*_ is shifted in the forward direction, ***n***⋅*δ****x***>0, in a thin layer of thickness ***n***⋅*δ****x*** the value of ℓ is changed from [ℓ]_+_ to [ℓ]_−_, hence the integral (3.4) has to be supplemented by the following correction term:

$$\int_{t_{1}}^{t_{2}}\sum_{n=1}^{N_{S}}\iint_{S_{n}}[[\ell ]]\text{n}\cdot \delta \text{x}\;dS\;dt.$$

Since ∇*φ*∥***n***, the identity ***n****δφ*=−(*δ****x***⋅∇*φ*)***n***=−(*δ****x***⋅***n***)∇*φ* results, leading to

$$\delta I=\int_{t_{1}}^{t_{2}}\sum_{n=1}^{N_{S}}\iint_{S_{n}}\left(\nabla \varphi \cdot \left[[\frac{\partial \ell }{\partial \nabla \varphi }-{\text{v}}_{s}\frac{\partial \ell }{\partial \dot{\varphi }}]\right]+[[\ell ]]\right)\text{n}\cdot \delta \text{x}\;dS\;dt.$$

Variation with respect to *φ* leads to the jump condition,

3.9 $$\nabla \varphi \cdot \left[[\frac{\partial \ell }{\partial \nabla \varphi }-{\text{v}}_{s}\frac{\partial \ell }{\partial \dot{\varphi }}]\right]=-[[\ell ]],$$

for the flux related to *φ* at the interface *S*_*n*_. From the physical viewpoint, this is related to the production of the associated integral quantity. Therefore, we call (3.9) the *production condition*.

There is an alternative way to derive the above production condition (3.9) along the lines of distribution theory sketched in §A.5 of appendix A, showing that the generalized form of the formalism can be understood in terms of standard Lagrange formalism.

## 4. Resulting equations of motion and matching conditions

### 4.1 Equations of motion

In §A.3 of appendix A the Euler–Lagrange equations resulting from variation with respect to the elementary fields are calculated. Based on this, in §A.4 the corresponding equations of motion are derived. As a result, the equations of motion for the observable fields (A.15), (A.20) are

4.1 ∇⋅u=0

and

4.2 $$D_{t}\text{u}=-\frac{\nabla p}{\varrho_{0}}+\nu \Delta \text{u}-\nabla V+{\text{f}}_{\text{n.e.}},$$

where ***f***_n.e._ is used as an abbreviation for

4.3 fn.e.:=−νω0{i ln ⁡χ¯χ[∇ trD_2+{Dt+∇⊗u}Δu]+{Dt+∇⊗u}[2D_Im∇χχ]}.

Obviously, by (4.1) the continuity equation is perfectly reproduced, whereas the equations of motion (4.2) differ from the original Navier–Stokes equations by some additional forces ***f***_n.e._. Since according to (4.3) the latter contain the complex field of thermal excitation, *χ*, the corresponding evolution equation (A.19),

4.4 $$D_{t}\chi +i\omega_{0}\chi =\frac{\nu }{2\bar{\chi }}\;\mathrm{tr}\;{\underline{D}}^{2},$$

has to be considered additionally in order to complete the set of equations.

Reconsidering the aforementioned hypothesis of Anthony [15] as well as Zuckerwar & Ash [14] to identify the additional forces occurring in the equations of motion as contributions due to a deviation from the thermodynamic local equilibrium, one would expect a limit case leading to the classical dynamics, as already discussed at the end of §(a). Indeed, according to (4.3) the extra forces are scaled down when increasing the parameter *ω*_0_ and the physical dimension of *ω*_0_ is a reciprocal of time, suggesting its interpretation as a relaxation rate. This interpretation is underpinned by considering the limit $\omega_{0}\to \infty$, leading to vanishing of the extra forces, ***f***_n.e._→0, and therefore to a full reproduction of the Navier–Stokes equations by means of (4.2). In this equilibrium limit, the evolution equation (4.4) becomes meaningless, since the field of thermal excitation does not appear in the equations of motion any more.

It is noteworthy that the limit $\omega_{0}\to \infty$ can be applied successfully to the equations of motion (4.2) on the one hand, reproducing Navier–Stokes equations, but cannot be applied directly to the Lagrangian (2.31) on the other hand, most likely because the mechanical equations (4.1) and (4.2) are decoupled from thermodynamics, whereas in the viscosity term of the Lagrangian (2.31) the occurring mechanical and thermodynamic degrees of freedom are strictly coupled.

For finite but sufficiently large values of *ω*_0_, the additional forces ***f***_n.e._→0 due to thermodynamic non-equilibrium remain small compared with viscous, external and pressure forces. According to (4.3), they consist of a factor $i\;\ln\;\sqrt{\bar{\chi }/\chi }$ expeditiously fluctuating between −*π* and *π*, of quadratic terms with respect to velocity gradients and of third-order derivatives of the velocity.

### 4.2 Matching conditions

As shown in §(b), variation with respect to the elementary fields, except for *χ*, induces matching conditions (3.7) at each interface. These are

4.5 $$\begin{array}{rl} \delta \Phi : & \;0=-\text{n}\cdot [[\varrho_{0}(\text{u}-{\text{v}}_{s})]], \end{array}$$

4.6 $$\begin{array}{rl} \delta \alpha : & \;0=0, \end{array}$$

4.7 $$\begin{array}{rl} \delta \beta : & \;0=-\text{n}\cdot [[\varrho_{0}\alpha (\text{u}-{\text{v}}_{s})]] \end{array}$$

4.8 $$\begin{array}{rl} \text{and}\;\delta \text{u}: & \;0=\varrho_{0}\frac{\nu }{i\omega_{0}}\text{n}\left[\left[\;\ln\;\sqrt{\frac{\bar{\chi }}{\chi }}\underline{D}\right]\right]. \end{array}$$

According to the first condition (4.5), the normal component of mass flux density has to be continuous, which physically corresponds to the conservation of the mass passing the interface. By inserting (4.5) into condition (4.7), it reduces to [[*α*]]=0, implying continuity of the Clebsch variable *α*. In order to understand the physics behind condition (4.8), we have to take into account that at each discontinuity the phase of the thermal excitation jumps from *π* to −*π* or vice versa. In any case, the sign of $\text{i}\;\ln\;\sqrt{\bar{\chi }/\chi }$ changes when turning from the back side to the front side of an interface. As a consequence, condition (4.8) entails

4.9 $$\text{n}{\left[\underline{D}\right]}_{-}+\text{n}{\left[\underline{D}\right]}_{+}=\text{0},$$

which implies a reversal of the direction of the shear rate vector $\underline{D}\text{n}$ at the inner boundary. Physically, this is associated with a slip occurring at the interface and can again be interpreted as a phenomenon due to a deviation from thermodynamic equilibrium.

### 4.3 Production condition and thermodynamic aspects

In order to apply formula (3.9) for the production condition, the complex field of thermal excitation has to be decomposed into modulus and phase according to $\chi =\sqrt{c_{0}T}\exp(-\text{i}\varphi )$, leading to the real-valued form

4.10 $$\ell =-\varrho_{0}\left[D_{t}\Phi +\alpha D_{t}\beta -\frac{c_{0}T}{\omega_{0}}D_{t}\varphi -\frac{{\text{u}}^{\;2}}{2}+c_{0}T+V-\frac{\nu }{\omega_{0}}S(\varphi )\;\mathrm{tr}\;{\underline{D}}^{2}\right]$$

of the Lagrangian (2.31), where *S* again denotes the sawtooth function. Then, the production condition reads:

4.11 $$\frac{\varrho_{0}}{\omega_{0}}\nabla \varphi \cdot [[c_{0}T(\text{u}-{\text{v}}_{s})]]=\varrho_{0}\frac{\nu }{\omega_{0}}[[S(\varphi )\;\mathrm{tr}\;{\underline{D}}^{2}]].$$

Despite the reversal of the shear rate tensor at the interfaces according to (4.9), its square, ${\underline{D}}^{2}$, remains continuous. Hence it follows that $[[S(\varphi )\;\mathrm{tr}\;{\underline{D}}^{2}]]=[[S(\varphi )]]\;\mathrm{tr}\;{\underline{D}}^{2}=2\pi \;\mathrm{tr}\;{\underline{D}}^{2}$, leading to:

4.12 $$\frac{1}{2\pi }\nabla \varphi \cdot [[c_{0}T(\text{u}-{\text{v}}_{s})]]=\nu \;\mathrm{tr}\;{\underline{D}}^{2}.$$

The above condition reveals a discontinuity in the flux of the inner energy and therefore the production of inner energy due to dissipation at the interfaces. The latter one can alternatively be related to the volume by estimating the gradient of the thermal phase as ∇*φ*≈(2*π*/*d*)***n***, where *d* denotes the distance between two interfaces. As a consequence, the inhomogeneity at the right-hand side of the balance (4.12) can, according to $\nu \;\mathrm{tr}\;{\underline{D}}^{2}\approx [[c_{0}T(\text{u}-{\text{v}}_{s})]]/d$, be re-interpreted as the mean production of inner energy related to the volume, at least in the sense of a statistical treatment.

Another source of inner energy production is already given by equation (A.21):

4.13 $$c_{0}{\text{D}}_{t}T=\nu \;\mathrm{tr}\;{\underline{D}}^{2},$$

which takes the form of a classical balance equation in continuum mechanics with a production rate $\nu \;\mathrm{tr}\;{\underline{D}}^{2}$ related directly to the volume. Compared with the classical theory of viscous flow [20,21], the production of inner energy due to dissipation is twice the value occurring in equation (4.13). Since, however, by (4.12) an additional production of inner energy at the inner boundaries is revealed, giving a contribution of the same amount as (4.13), we reason that the total production of inner energy is in accordance with classical theory.

The question arises as to whether the occurrence of discontinuous interfaces inside the fluid flow is an artefact of the model or if such phenomena really exist on a microscopic scale. Although a final answer to this question cannot be provided since knowledge about the processes occurring in a fluid flow on the micro-scale is restricted at the present time, it can be conjectured what kind of effect slight changes of the model may cause. At least the model established in this paper accurately recovers the physics on a macroscopic scale, and there are dissipative phenomena with entropy production at discontinuous surfaces known for a long time: we refer in particular to the classical theory of shock waves [20] where entropy production at discontinuous surfaces is provoked by a rapid compression of gas. Here, the discontinuous phenomena are not compression waves but can be considered as ‘slip waves’ which becomes apparent by (4.9).

Within this context, it is also of particular interest how the physics would be affected by a change of branch cut for the complex logarithm: in §(b) the standard branch cut along the negative real axis has been used, leading to values of $\;\ln\;\sqrt{\bar{\chi }/\chi }$ between −i*π* and i*π*. One consequence of this is that over time the fluctuating forces (4.3) occurring in the equations of motion statistically result in zero by averaging. If an alternative branch cut for the complex logarithm is considered, say e.g. a cut at arguments of the complex number if *a*−*π*, the positions of the discontinuous surfaces are shifted and the values of the complex logarithm go from i*a*−i*π* to i*a*+i*π*. The first effect, the shift of the discontinuous surfaces, could be compensated for by applying the gauge transformation χ→χexp⁡(−ia) on the field of thermal excitation, whereas the second effect causes a change of the fluctuating forces (4.3) according to:

$${\text{f}}_{\text{n.e.}}⟶{\text{f}}_{\text{n.e.}}+\frac{\nu a}{\omega_{0}}[\nabla \;\mathrm{tr}\;{\underline{D}}^{2}+\{D_{t}+\nabla ⊗\text{u}\}\Delta \text{u}].$$

The extra term on the right hand side vanishes in the limit $\omega_{0}\to \infty$; however, for finite values of *ω*_0_ it gives an additional contribution to the equations of motion. By averaging again, this additional term does not tend to zero which is a valid physical argument for the use of the standard branch cut along the negative real axis (i.e. *a*=0).

## 5. Discussion

Through the detailed analysis in §4, it has been demonstrated that the dynamics resulting from the Lagrangian (2.31) can self-consistently be interpreted as an extension of the classical theory towards processes beyond thermodynamic local equilibrium. A reproduction of the classical theory is reached by applying the limit $\omega_{0}\to \infty$ for the relaxation rate to the equations of motion, a procedure that was not possible for the earlier suggested Lagrangian (2.4). In the following, some further indications are given in order to confirm the non-equilibrium assumption.

As already stated in §(a) in the context of the Lagrangian (2.4), a striking non-classical feature is the difference between the momentum density and the mass flux density, namely the quasi-momentum density p*=***p***−*ϱ****u***. In the context of the Lagrangian (2.31), the mass flux density is given via (A.18), whereas the momentum density is obtained as a canonical Noether observable [6], giving: p=ϱ0[∇Φ+α∇β+(1/ω0)Im(χ¯∇χ)]. Hence, there is again a non-vanishing quasi-momentum density,

5.1 $${\text{p}}^{*}=\varrho_{0}\frac{\nu }{\omega_{0}}\nabla \cdot \left[i\;\ln\;\sqrt{\frac{\bar{\chi }}{\chi }}2\underline{D}\right],$$

which in contrast to the quasi-momentum density (2.8) resulting from the Lagrangian (2.4) tends to zero for the limiting case $\omega_{0}\to \infty$. This is again in accordance with classical continuum theory.

**Figure 6.**
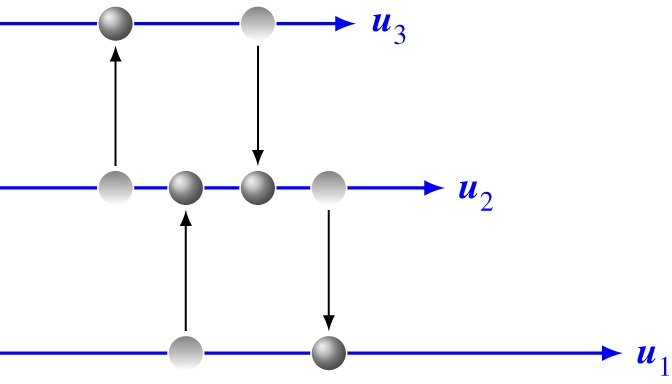
A simple microscopic model for viscosity, based on migration of particles between neighboured fluid layers by Brownian motion.

Following the suggestion in previous work [6,12,13] that the quasi-momentum takes into account contributions to the momentum beyond the scope of the continuum hypothesis on a molecular scale, e.g. Brownian motion, a possible physical interpretation of the quasi-momentum density (2.8) is based on the elementary mechanism of viscosity, namely the transport of momentum by Brownian motion crosswise to the flow direction, see figure 6. The viscosity of a fluid is usually explained on a molecular scale by an exchange of particles between neighbouring fluid layers by Brownian motion of the molecules, by which a diffusion of momentum is induced. From the continuum viewpoint, the migrating particles responsible for the diffusive momentum flux are ‘quasi-particles’, associated with an additional contribution to the momentum density. Hence, the quasi-momentum density can also be considered as a measure for the deviation from thermodynamic equilibrium.

Note that the interpretation of the additional terms in the equations of motion as physical non-equilibrium effects on a microscopic scale is also consistent with the weak violation of the continuum hypothesis imposed by the discontinuous Lagrangian (2.31). Regardless, the violation of the continuum hypothesis vanishes in the limit $\omega_{0}\to \infty$ for the relaxation rate: for increasing *ω*_0_, the discontinuities are decreasing and for very large *ω*_0_ they are physically reduced to fluctuations on a micro-scale in accordance with classical theory.

## 6. Conclusion and outlook

Based on an analogy between quantum mechanics and fluid mechanics, the formerly proposed Lagrangian (2.4) has been refined, leading to the discontinuous Lagrangian (2.31). By a careful analysis, it is proved that the dynamics resulting from Hamilton's principle based on (2.31) can consistently be interpreted as a generalization of the theory of viscous flow towards thermodynamic non-equilibrium, with a recovery of the classical Navier–Stokes equations and the balance of inner energy when applying the limit $\omega_{0}\to \infty$ to the resulting equations of motion. As a striking feature, the application of the limit $\omega_{0}\to \infty$ directly to the Lagrangian (2.31) fails. Hence, at least an indication is given that a variational formulation of viscous flow cannot be achieved using a continuous Lagrangian.

Although for large *ω*_0_ the discontinuities are physically reduced to fluctuations on a micro-scale, it would be of great interest for future work to explore further the dynamics of the discontinuous interfaces and the induced physical effects beyond thermodynamic equilibrium for various flow geometries.

As already mentioned in the Introduction, a striking feature for variational formulations in continuum mechanics is the necessity for the use of potentials in general [6]. Since the Euler–Lagrange equations resulting from the two Lagrangians (2.4) and (2.31), the latter one is derived in §A.3 of appendix A, can also be interpreted as a *first integral* of the equations of motion, the use of potential fields seems also to be inevitable for the construction of first integrals of the equations of motion in fluid mechanics, see for example the attempts of He [28,29] for finding a potential representation of the velocity for two-dimensional incompressible and inviscid flow. For viscous flow, a first integral approach has been established using a generalized form of the Clebsch transformation [30], and based on the use of potentials [31,32], methodically different from the Clebsch transformation but with some interesting parallels to the approach given in this paper. The latter will be analysed in forthcoming papers.

By evaluating the dynamics induced by the Lagrangian (2.31), it has been demonstrated how Lagrange formalism applies to physical problems with discontinuities. Independent of the particular problem of viscous flow, the general formalism specified in §3 may also be a valuable mathematical tool for embedding various discontinuous phenomena into Lagrange formalism, like for example phase boundaries between non-mixable fluids, propagating shock waves in gaseous media, flame fronts, detonation shocks and also interfaces in solids like micro-cracks [33] and grain boundaries. Discontinuities also occur in some optimum control problems [34]. The extended formalism can be used for finite-element simulations of such phenomena without the imperative of considering the related matching conditions explicitly, since they result automatically from the respective Lagrangian. It is therefore realistic to expect an improvement of, for example, numerical algorithms.

## Data accessibility

This is a pure theoretical work without experimental or numerical data. The analytical calculations performed are transparently given in the accompanying appendix.

## Appendix A. Calculations

**A.1 Symmetries and associated Noether balances of the Lagrangian (2.4)**

In Scholle [6], a general analysis is provided concerning the analytical structure of Lagrangians in continuum theories fulfilling invariance with respect to the full Galilei group. In the same paper, a general scheme for Lagrangians is constructed. Using Noether's theorem, canonical formulae give rise to the identification of the relevant observable fields like mass density and flux density, momentum density, stress tensor, energy density and Poynting vector.

Subsequently, the analysis given in Scholle [6] is rigorously applied to the Lagrangian (2.4): for simultaneous invariance with respect to time and space translations and Galilei boosts, a collective symmetry criterion, the *duality criterion*,

A 1 $$\ell \left(\Psi^{i},\overset{\circ }{\Psi^{i}},\nabla \Psi^{i}+\frac{1}{t}{\text{K}}^{i}(\Psi^{j})\right)=\ell (\psi^{i},{\dot{\psi }}^{i},\nabla \psi^{i}),$$

has to be fulfilled where

$$\begin{array}{rl} \dot{\psi } & =\frac{\partial \psi }{\partial t},\\ \overset{\circ }{\Psi } & =\left\{\frac{\partial }{\partial t}+\nabla \zeta \cdot \nabla \right\}\Psi \\ \mathrm{and}\;\zeta & =\frac{{\text{x}}^{2}}{2t} \end{array}$$

are the conventional time derivative, the dual time derivative and the generating field, respectively, and

A 2 $$\psi^{i}=K^{i}(\Psi^{j},\zeta ,\nabla \zeta )$$

and

A 3 $${\text{K}}^{i}(\Psi^{j})=\lim_{\zeta ,\nabla \zeta \to 0}\frac{\partial K^{i}}{\partial (\nabla \zeta )}$$

are the dual transformation and the corresponding infinitesimal generator. In the case of the present Lagrangian (2.4), the dual transformation takes the form

A 4 $$\begin{array}{rlr} \text{u} & =\text{U}+\nabla \zeta , & \;\varphi =\Phi +\zeta ,\\ \alpha & =A & \;\beta =B,\\ \varrho & =P & \;s=S\\ \text{and}\;\vartheta & =\Theta \end{array}\}$$

fulfilling criterion (A.1) as required. One consequence of it is that the mass balance,

A 5 $$\frac{\partial \varrho }{\partial t}+\nabla \cdot (\varrho \text{u})=0,$$

is automatically fulfilled and *ϱ****u*** is identified as the mass flux density. In the following, we skip the computation of canonical stress tensor, energy density and Poynting vector form Noether's theorem and focus on the canonical momentum density which takes the form [6]

A 6 $$\text{p}=-\frac{\partial \ell }{\partial \dot{\psi^{i}}}\nabla \psi^{i}=\varrho [\nabla \varphi +\alpha \nabla \beta -s\nabla \vartheta ],$$

and need not be identical to the mass flux density *ϱ****u***. Since the dual transformation formula (A.4) contains a real ∇*ζ*-dependence, the relation

A 7 $$\text{p}=\varrho \text{u}+{\text{p}}^{*}$$

is given with the *quasi-momentum density*:

A 8 $${\text{p}}^{*}=-\frac{\partial }{\partial t}\left[\frac{\partial \ell }{\partial \dot{\psi^{i}}}{\text{K}}^{i}\right]-\nabla \cdot \left[\frac{\partial \ell }{\partial \nabla \psi^{i}}{\text{K}}^{i}\right]=-\nabla \cdot \left(\frac{\vartheta }{T}[2\eta \underline{D}+\eta^{\prime }(\nabla \cdot \text{u})\underline{1}]\right).$$

Hence, a striking feature of the Lagrangian (2.4) is a difference between mass flux density and momentum density which is in contrast to classical continuum mechanics.

**A.2 Steady solution of (2.9)–(2.11) for Poiseuille flow**

Considering the flow geometry ***u***=*u*(*y*)***e***_*x*_ and assuming time-independent thermasy *ϑ*, the evolution equation (2.11) takes the form

A 9 $$u(y)\frac{\partial }{\partial x}\left(\frac{\vartheta }{T}\right)=1,$$

which obviously implies the general solution (2.15). Considering (A.9), equations (2.9) read

$$\begin{array}{rl} \frac{\nabla p}{\nu \varrho_{0}} & =\left\{u\frac{\partial }{\partial x}+u^{\prime }e_{y}⊗e_{x}\right\}\left[u^{\prime }\left(e_{x}⊗e_{y}+e_{y}⊗e_{x}\right)\nabla \left(\frac{\vartheta }{T}\right)+\frac{\vartheta }{T}\Delta \text{u}\right]-\frac{1}{2}{u^{\prime }}^{2}\nabla \left(\frac{\vartheta }{T}\right)\\ & =uu^{\prime }\frac{\partial }{\partial x}\left\{e_{x}\frac{\partial }{\partial y}+e_{y}\frac{\partial }{\partial x}\right\}\frac{\vartheta }{T}+u^{\prime \prime }e_{x}+\left[{u^{\prime }}^{2}\frac{\partial }{\partial y}\left(\frac{\vartheta }{T}\right)+u^{\prime }u^{\prime \prime }\frac{\vartheta }{T}\right]e_{y}-\frac{1}{2}{u^{\prime }}^{2}\nabla \left(\frac{\vartheta }{T}\right)\\ & =\left[u^{\prime \prime }-\frac{3{u^{\prime }}^{2}}{2u}\right]e_{x}+\frac{\partial }{\partial y}\left[\frac{{u^{\prime }}^{2}}{2}\frac{\vartheta }{T}\right]e_{y}. \end{array}$$

Taking the component in the *y*-direction, it follows that

$$p=\eta \frac{\vartheta }{T}\frac{{u^{\prime }}^{2}}{2}+f_{2}(x),$$

for the pressure (*η*=*ϱ*_0_*ν*) with integration function *f*_2_(*x*). By inserting this into the equation for the *x*-direction, we obtain

$$\frac{f_{2}^{\prime }(x)}{\eta }=u^{\prime \prime }-2\frac{{u^{\prime }}^{2}}{u}.$$

Since the left hand side of the above equation depends on *x* only and the right hand on *y* only, both sides are constant, i.e.

$$\frac{f_{2}^{\prime }(x)}{\eta }=-K$$

and

$$u^{\prime \prime }-2\frac{{u^{\prime }}^{2}}{u}=-K,$$

with *K*>0. Hence, the pressure is evaluated as

A 10 $$p=p_{0}-\eta Kx+\eta \frac{\vartheta }{T}\frac{{u^{\prime }}^{2}}{2},$$

whereas the velocity profile has to fulfil the nonlinear ODE

A 11 $$uu^{\prime \prime }-2{u^{\prime }}^{2}+Ku=0.$$

Via the substitution *g*(*y*)=1/*u*(*y*), equation (A.11) simplifies to the second-order ODE *g*′′−*Kg*^2^=0. After multiplication with *g*′ the latter becomes integrable,

$$\frac{d}{dy}\left[\frac{{g^{\prime }}^{2}}{2}-K\frac{g^{3}}{3}\right]=0,$$

implying the first-order ODE *g*′^2^−2*Kg*^3^/3=*C*. The integration constant, *C*, is evaluated at the middle of the channel, *y*=*h*/2, where the velocity takes its maximum, *u*(*h*/2)=*u*_max_, and therefore *u*′(*h*/2)=0, implying *C*=−2*K*/(3*u*^3^_max_). Hence, after re-substituting *u*=1/*g*,

$$u^{\prime }=\pm \sqrt{\frac{2K}{3}\left[1-\frac{u^{3}}{u_{\text{max}}^{3}}\right]u}$$

results as a nonlinear first-order ODE for the velocity profile, with the positive sign valid for 0≤*y*<*h*/2 and the negative sign for *h*/2<*y*≤*h*. Using separation of variables, the above ODE can be solved, with the solution for *y*≤*h*/2 implicitly given as

A 12 $$y=\sqrt{\frac{2u}{K}}{}_{2}F_{1}\left(\frac{1}{6},\frac{1}{2};\frac{7}{6};\frac{u^{3}}{u_{\text{max}}^{3}}\right)$$

and

A 13 $$u_{\text{max}}=\frac{9\Gamma {\left(2/3\right)}^{2}\Gamma {\left(5/6\right)}^{2}}{8\pi^{3}}Kh^{2},$$

with Gaussian hypergeometric function _2_*F*_1_.

**A.3 Euler–Lagrange equations of the Lagrangian (2.31)**

The Lagrangian (2.31) is based on the following fields: the velocity ***u***, the three Clebsch variables *Φ*, *α*, *β* and the complex field of thermal excitation *χ*. Subsequently, the associated Euler–Lagrange equations are computed according to (3.3). First, variation with respect to the Clebsch variable *Φ* delivers the continuity equation

A 14 ∇⋅u=0.

As a consequence, the identity ∇⋅(*ξ****u***)=***u***⋅∇*ξ*, which is fulfilled for any field *ξ*, is considered subsequently. Next, variation with respect to the two remaining Clebsch variables *α* and *β* lead to the transport equations

A 15 $$D_{t}\beta =0$$

and

A 16 $$D_{t}\alpha =0,$$

after simple mathematical manipulation. By variation with respect to the velocity ***u***,

A 17 ϱ0u+ϱ0νω0∇⋅[i ln ⁡χ¯χ2D_]=ϱ0[∇Φ+α∇β+1ω0Im(χ¯∇χ)]

is obtained. Finally, the Euler–Lagrange equation related to the variation with respect to $\bar{\chi }$ leads to the evolution equation

A 18 $$D_{t}\chi +i\omega_{0}\chi =\frac{\nu }{2\bar{\chi }}\mathrm{tr}{\underline{D}}^{2},$$

for the thermal excitation. Variation with respect to $\bar{\chi }$ delivers the complex conjugate of (A.19).

**A.4 Equations of motion**

The Euler–Lagrange equations (A.16)–(A.19) are a first integral of the equations of motion, i.e. the latter can be obtained from their derivations as follows: considering the identities

Dt[∇Φ+α∇β+Imχ¯∇χω0]=∇[DtΦ+αDtβ+Imχ¯Dtχω0]−∇⊗u[∇Φ+α∇β+Imχ¯∇χω0]+Dtα∇β−Dtβ∇α−2ω0Im(Dtχ∇χ¯),∇⋅[i ln ⁡χ¯χ2D_]=2D_Im∇χχ+i ln ⁡χ¯χ[∇(∇⋅u)+Δu],iDt ln ⁡χ¯χ=Im(Dtχχ),

and the Euler–Lagrange equations (A.15)–(A.19), the material time derivative of (A.18) reads

A 19 Dtu−νΔu+νω0[i ln ⁡χ¯χDtΔu+Dt(2D_Im∇χχ)]=∇[DtΦ+αDtβ+Imχ¯Dtχω0]−∇⊗u[u+νω0∇⋅(i ln ⁡χ¯χ2D_)]−2ω0Im[(ν2χ¯trD_2−iω0χ)∇χ¯]=−1ϱ0∇p−∇V−iνω0 ln ⁡χ¯χ∇trD_2−νω0∇⊗u[2D_Im∇χχ+i ln ⁡χ¯χΔu],

where the pressure *p* is according to

p:=−ϱ0[DtΦ+αDtβ+Imχ¯Dtχω0−u22+χ¯χ+V−νiω0 ln ⁡χ¯χtrD_2]=ℓE.L.,

given as the Lagrangian evaluated for real processes, as is well known from the classical literature [35]. Finally, a balance for the inner energy, $c_{0}T=\bar{\chi }\chi$ is derived from (A.19) according to:

A 20 $$c_{0}{\text{D}}_{t}T={\text{D}}_{t}(\bar{\chi }\chi )=\bar{\chi }{\text{D}}_{t}\chi +\chi {\text{D}}_{t}\bar{\chi }=\nu \mathrm{tr}{\underline{D}}^{2}.$$

**A.5 Derivation of the production condition (3.9) using distributions**

Within the theory of distributions, a very elegant way is provided by which the generalized formalism for discontinuous Lagrangians can be understood in terms of conventional Lagrange formalism. Under the same assumptions made in §3, by

A 21 $$\ell_{\text{c}}(\cdots ,\varphi ):=\ell (\cdots ,\varphi )-\sum_{n=1}^{N_{S}}[[\ell (\cdots ,\varphi_{n})]]H(\varphi -\varphi_{n})$$

a continuous reference Lagrangian ℓ_c_ is defined, where H(x) is the Heaviside function giving 0 for *x*<0 and 1 for *x*≥0. Equation (A.22) defines a decomposition of the entire Lagrangian into a continuous part and a sum of discontinuities; the derivative of it with respect to *φ* gives

A 22 $$\frac{\partial \ell }{\partial \varphi }=\frac{\partial \ell_{\text{c}}}{\partial \varphi }+\sum_{n=1}^{N_{S}}[[\ell (\cdots ,\varphi_{n})]]\delta (\varphi -\varphi_{n}),$$

where *δ*(*x*) denotes Dirac's delta function. Using this, the Euler–Lagrange expression *EL*_*N*_ defined according to (3.3) with respect to *ψ*_*N*_=*φ* can be defined across the entire domain, leading to

$$\begin{array}{rl} {\mathrm{EL}}_{N} & =\frac{\partial \ell }{\partial \varphi }-\frac{\partial }{\partial t}\left(\frac{\partial \ell }{\partial \dot{\varphi }}\right)-\nabla \cdot \left(\frac{\partial \ell }{\partial \nabla \varphi }\right)\\ & =\underset{:={\mathrm{EL}}_{N}^{\text{c}}}{\underbrace{\frac{\partial \ell_{\text{c}}}{\partial \varphi }-\frac{\partial }{\partial t}\left(\frac{\partial \ell_{\text{c}}}{\partial \dot{\varphi }}\right)-\nabla \cdot \left(\frac{\partial \ell_{\text{c}}}{\partial \nabla \varphi }\right)}}+\sum_{n=1}^{N_{S}}[[\ell (\cdots ,\varphi_{n})]]\delta (\varphi -\varphi_{n}). \end{array}$$

As a consequence of the above decomposition, the first term in equation (3.5) for *ψ*_*N*_=*φ* results in

A 23 $$\int_{t_{1}}^{t_{2}}\sum_{n=0}^{N_{S}}{∭}_{V_{n}}{\mathrm{EL}}_{N}\delta \varphi \;dV\;dt=\int_{t_{1}}^{t_{2}}{∭}_{V}{\mathrm{EL}}_{N}^{\text{c}}\delta \varphi \;dV\;dt+\int_{t_{1}}^{t_{2}}\sum_{n=0}^{N_{S}}{∭}_{V}[[\ell ]]\delta \varphi \delta (\varphi -\varphi_{n})\;\text{d}V\;dt.$$

In order to evaluate the integrals at the right hand with delta functions, a representation of d*V* in terms of local coordinates, d*V* =d*ξ* d*S* is used where d*S* is the surface element of the interface *S*_*n*_ and d*ξ* is related to the direction perpendicular to *S*_*n*_. By the substitution d*φ*=|∇*φ*| d*ξ*, the identity

$${∭}_{V}[[\ell ]]\delta \varphi \delta (\varphi -\varphi_{n})\;\text{d}V={∭}_{V}\frac{[[\ell ]]\delta \varphi }{|\nabla \varphi |}\delta (\varphi -\varphi_{n})\;\text{d}\varphi \;\text{d}S=\iint_{S_{n}}\frac{[[\ell ]]\delta \varphi }{|\nabla \varphi |}\;dS$$

is obtained, giving (A.24) the more convenient form

A 24 $$\int_{t_{1}}^{t_{2}}\sum_{n=0}^{N_{S}}{∭}_{V_{n}}{\mathrm{EL}}_{N}\delta \varphi \;dV\;dt=\int_{t_{1}}^{t_{2}}{∭}_{V}{\mathrm{EL}}_{N}^{\text{c}}\delta \varphi \;dV\;dt+\int_{t_{1}}^{t_{2}}\sum_{n=0}^{N_{S}}\iint_{S_{n}}\frac{\left[[\ell ]\right]}{|\nabla \varphi |}\delta \varphi \;dS\;dt.$$

Finally, considering the identity (3.6) and the vanishing of *δφ* at *t*=*t*_1,2_, the variation of the action integral, (3.5), turns out to be

A 25 $$\delta I=\int_{t_{1}}^{t_{2}}{∭}_{V}{\mathrm{EL}}_{i}^{\text{c}}\delta \varphi \;dV\;dt+\int_{t_{1}}^{t_{2}}\sum_{n=0}^{N_{S}}\iint_{S_{n}}\left\{\text{n}\cdot \left[[\frac{\partial \ell }{\partial \nabla \psi_{i}}-{\text{v}}_{s}\frac{\partial \ell }{\partial {\dot{\psi }}_{i}}]\right]+\frac{\left[[\ell ]\right]}{|\nabla \varphi |}\right\}\delta \varphi \;dS\;dt,$$

which apparently implies, next to the well-known Euler–Lagrange equation with respect to *φ*, the production condition (3.9).
